# Growth of bubbles in liquid

**DOI:** 10.1186/s13065-015-0127-y

**Published:** 2015-09-21

**Authors:** Boris M. Smirnov, R. Stephen Berry

**Affiliations:** Joint Institute for High Temperatures, Izhorskaya 13/19, Moscow, 127412 Russia; Department of Chemistry, University of Chicago, 929 East 57th Street, Chicago, IL 60637 USA

**Keywords:** Bubbles in liquid, Oxygen in water, Floating-up of bubbles, Growth of bubbles

## Abstract

**Background:**

Evolution of a gas injected in a liquid is analyzed using the example of the behavior of oxygen molecules in water in which bubbles of gas molecules grow slowly by attachment of gas molecules to bubbles, the bubbles then associate and finally flow up to the liquid–gas interface and pass into the gas phase.

**Results:**

Two methods are considered for gas injection in a liquid, insertion of individual molecules and injection of small gas bubbles via gas penetration through a porous material. The behavior of oxygen bubbles in water and growth of those bubbles is analyzed. Subsequently, these grown bubbles float up and disappear, or reach the water boundary as a result of turbulent motion of the liquid.

**Conclusions:**

It is shown that measurement of the size distribution function of micron-size bubbles in various regions of the water container allows one to establish the flow current lines on the basis of the theory of bubble growth.

**Graphical abstract:**
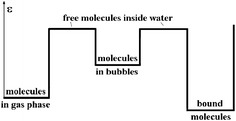
Schematic diagram of energies of molecules in the gas phase, in bubbles and bound to solvent

## Background

**Fig. 1:**
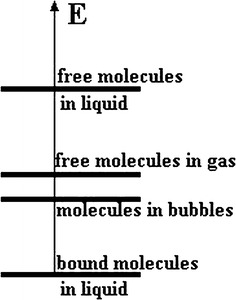
Energy levels for gaseous molecules located *inside* and *outside* a liquid

If gas molecules are located in a simple liquid, they may be found in three states. The molecule may be bounded in knots of the liquid like a molecule in a knot of crystal lattice where the transition of such a molecule a neighboring knot occurs relatively alowly. In this state a gas is dissolved in the liquid, and the solution of such molecules is a stable state of this disperse system. But the concentration of bound gas molecules is restricted by the number of knots in the liquid where these molecules are bound. If the concentration of gas molecules exceeds their maximum concentration at a given temperature, the excess of gas molecules leaves the gas under thermodynamic equilibrium. But a typical time of establishment of this equilibrium may be long because excess molecules leave the liquid through the interface, and gas molecules reach it due to diffusion. We below consider a nonequilibrium state of the gas-liquid system in which the concentration of gas molecules exceeds the saturated level. On the way to leave the liquid, free gas molecules join into bubbles, and the floating-up of gas bubbles accelerates establishment of thermodynamic equilibrium. The floating-up takes place to the interface, if a liquid borders with a free gas, or to walls of a tube, if the liquid flows inside this tube. The joining of bubbles in the course of their residence inside the liquid is important, and because a typical floating-up time depends on bubble size, kinetics of bubble growth is important for this. Thus, our goal is the analysis of kinetics of bubble growth together with floating-up of bubbles. In this analysis we will be guided by water as a liquid and oxygen as a gas. We also use an analogy of bubble growth in a liquid with the process of cluster growth in a gas.

## Introduction

In this paper we consider the character of evolution of a gas which penetrates through a porous medium into an open, liquid-filled reservoir. At low flow rates, the gas forms bubbles in the liquid which subsequently float to the upper liquid surface and disappear into air. These bubbles may also be formed in natural processes, as occurs in the case of methane produced by putrefaction processes in marsh. Photosynthesis processes in water lead to oxygen formation, which may have the form of bubbles and in that condition, influence the existence of living organisms in water. Apart from natural processes, such processes may be used to saturate liquids by gases, e.g., filling of an aquarium by oxygen. One of properties of a disperse matter consisting of a liquid containing gaseous bubbles is the low electric field strength of electric breakdown of this system compared to a pure liquid. This may be used in purification of a water flow. Because the gasification process of liquids is slow, the energetics of this process are low and such processes are available in reality.

Formation of gas bubbles in a liquid is possible for any liquid system. For example, ageing of some liquids results from chemical reactions which are accelerated due to bubble formation. In particular, oil degradation in a power transformer comes from oil decomposition with formation of gaseous bubbles. Consequently those bubbles are the matter through which electric breakdown is realized in power units. One more example of bubble formation proceeds in the course of solidification. If an alloy is formed by cooling of a liquid, gaseous bubbles may remain inside it and determine the subsequent strength of this material. Thus, we have a large number of situations in which gaseous bubbles are formed and develop in a liquid. Then bubble growth may be result both from attachment of individual molecules to bubbles and by coagulation of bubbles. We here consider the kinetics of bubble growth in a liquid if a gas is dissolved or is injected into the liquid.

In considering the kinetics of evolution of gases in liquids through formation of bubbles which eventually float up, we will be guided by air bubbles located in water since considerable information about this system is available that allows us to find realistic parameters of this process. So, let us consider a general situation of air dissolving in water either in a stagnant basin or in a flowing water reservoir. As a result, the air initially accumulates in water in the form of separate molecules. The same takes place if oxygen is formed in water in the photosynthesis process, and the process of oxygen accumulation proceeds continuously during a long time interval. When the amount of a dissolved gas exceeds the limit of its water solubility, the gas molecules join in aggregates which form bubbles in the water. These bubbles grow as a result of processes of coagulation and coalescence and simultaneously they are floating up. In the end we obtain a steady-state picture in which new portions of molecules are injected in water, form bubbles, and leave as a result of bubbles flowing up.

## Oxygen in water

In considering the equilibrium between a gas in a liquid we will be guided by oxygen molecules in water, or by air molecules if we assume the behavior of nitrogen molecules to be analogous to that of oxygen molecules. There are three forms for oxygen molecules in water, and energetics of these forms is represented schematically in Fig. 1. Dissolved or bound oxygen molecules are found in thermodynamic equilibrium with oxygen molecules of air which borders with water. In particular, at a temperature of $T=298K$, the maximum amount of dissolved oxygen is 0.0089 g/l [1]. This corresponds to a partial pressure $p_s$ of the saturated amount of oxygen of $p_s(O_2)=5.2$ Torr or its concentration $c_{sat} \approx 5\times 10^{-6}=5$ ppm (the ratio of the number of dissolved oxygen molecules to the number of water molecules in a given volume). According to the Langmuir theory [2], one can introduce an effective number density of active knots $N_k$ for location of dissolved oxygen molecules at a given pressure. Then the number density of dissolved oxygen molecules $N(O_2)$ at a given temperature *T* is determined by formula

1 $$\begin{aligned} N(O_2)=N_k \exp \left( - \frac{\Delta \varepsilon }{T}\right) , \end{aligned}$$

where $\Delta \varepsilon$ is the binding energy of an oxygen molecule with an active knot. This formula holds true if the number density of free oxygen molecules outside water is large compared to the number density of dissolved molecules $N(O_2)$. Based on measurements [1], one can find values of the parameters of formula (1); that is,

2 $$\begin{aligned} N_k &\approx 4\times 10^{17}\,\mathrm{cm}^{-3},\nonumber\\ \Delta \varepsilon &=(1900 \pm 100)K=(0.16 \pm 0.01)eV. \end{aligned}$$

Note that the concentration of knots which can capture oxygen molecules is equal now to $c_k=12$ ppm [$c_k=N_k/N(H_2O)$, where $N(H_2O)$ is the number density of water molecules] and relates to atmospheric pressure. In particular, at the temperature $T=300$ K the number density $N_d$ of dissolved molecules and their concentration $c_d$ in water are equal

3 $$\begin{aligned} N_d=7\times 10^{14}\,\mathrm{cm}^{-3},\quad c_d=(22 \pm 7)\times 10^{-9}. \end{aligned}$$

The parameters of dissolved oxygen in accordance with formula (2) correspond to thermodynamic equilibrium. If the concentration of oxygen molecules exceeds the saturated value $c_{sat}$, the excess oxygen leaves the water through time. But if there is a source of oxygen injection into the water, a new equilibrium is formed where, together with the dissolved oxygen molecules, oxygen may also be found in the form of bubbles (see Fig. 1). In this case individual oxygen molecules may leave water by direct diffusion to the surface, but also can attach to bubbles. In turn, the bubbles can leave water via the interface both by diffusion and by flowing up; also bubbles may grow as a result of association of individual gas molecules. The goal of this paper is to analyze kinetics of bubble growth together with the escape of oxygen through the interface.

There are three methods to inject gas into a liquid. In the first case, individual molecules are introduced into the liquid, as for example when oxygen is generated in water via photosynthesis by aquatic plants. This generalizes to other chemical processes in a liquid. A second method results from a gas passing into a liquid through a porous material, as in experiment [3], in which air penetrates into water through a modified silica gel. This method is realized in the nature when, for example, methane forms in putrefaction processes, and passes into water in marshes. In the third method, a gas and liquid mix at their interface, as in turbulent flow of a stream or river, and this method requires a high power.

We next consider the regime of kinetics of undissolved oxygen, consisting principally of bubbles in water. The pressure inside a bubble of radius *r* is given by the Laplace formula [4]

4 $$\begin{aligned} p=p_{ex}+\frac{2\alpha }{r}, \end{aligned}$$

where $p_{ex}$ is the external pressure acting on a liquid, $\alpha$ is the surface tension that has the value $\alpha =72$ dyn/cm [5] for water at room temperature. This gives the number density *N* of air molecules inside a bubble of a radius *r*

5 $$\begin{aligned} N=N_o\left( 1+\frac{r_o}{r}\right) , \end{aligned}$$

and the parameters of this formula at pressure $p=1$ atm and room temperature are: $N_o = 2.46\times 10^{19}\,\mathrm{cm}^{-3},r_o=1.44\,\upmu \mathrm{m}$. In particular, from this it follows that a bubble with a radius of $r=1\,\upmu \mathrm{m}$ under the above conditions contains $n=2.5\times 10^8$ molecules.

## Thermodynamics of liquid with gas inside it

We below consider evolution of the system consisting of a liquid which borders with a gas and an equilibrium is established in the liquid–gas system through the interface. Let us consider this equilibrium for the above example of water as a liquid and oxygen as a gas, where water is located in a container and oxygen is placed over water. This equilibrium determines that a fraction of oxygen molecules are dissolved in water, i.e. these molecules are found in bound states in knots between water molecules. Such an equilibrium is described by formula (1) and oxygen molecules may be found in the lower and upper states of Fig. 1. But the establishment of this equilibrium is quite slow. Indeed, let us take a typical laboratory distance $L \sim 1$ cm from an oxygen molecule to interface, and an excess molecule reach the interface during a time $\tau = L^2/[2D(O_2)] \sim 10$ h, where the diffusion coefficient of oxygen molecules in water at room temperature is $D(O_2)=2.4\times 10^{-5} \,\mathrm{cm}^2/\mathrm{s}$ [5].

Let us consider an equilibrium where free oxygen molecules are injected in water, as takes place due to the photosynthesis processes, and obtain the above criterion of smallness of the number density of free oxygen molecules compared with that in bubbles. Let the loss of free oxygen molecules inside water be connected with their attachment to bubbles. The rate of molecule attachment to a bubble of a radius *r* is given by the Smoluchowski formula [6]

6 $$\begin{aligned} \nu _{at}=4\pi D(O_2)rN_p, \end{aligned}$$

where $N_p=N_b/n$ is the number density of bubbles, so that $N_b$ is the number density of oxygen molecules in bubbles, and *n* is the average number of bubble molecules. Evidently, the regime under consideration, where free oxygen molecules first attach to bubbles and then leave water in the form of bubble, requires the criterion $\nu _{at} \tau \gg 1$ or

7 $$\begin{aligned} 2\pi L^2rN_p \gg 1. \end{aligned}$$

In particular, for a typical bubble size $r=1\,\upmu \mathrm{m}$ this criterion gives

8 $$\begin{aligned} L^2N_b \gg 4\times 10^{11}\,\mathrm{cm}^{-1}. \end{aligned}$$

Evidently, bubbles are of importance for the behavior of the disperse system, if the number density of molecules in bubbles $N_b$ exceeds significantly the number density $N_d$ of molecules bonded inside water. From formula (3) it follows that in this case criterion (8) holds true. Note that in this consideration we assume a typical time of transition from free oxygen molecules to a bubble inside water to be brief compared to a typical lifetime of oxygen bubbles in water, and therefore the number density of oxygen molecules in bubbles exceeds significantly the current number density of free oxygen molecules inside water.

From this analysis one can conclude that thermodynamic equilibrium applies for separated liquid and gas and gas molecules dissolved in the liquid. This equilibrium is described by formula (1) in the limit under consideration. A liquid with a dissolved gas and gas bubbles inside it corresponds to a metastable state of this system which tends to thermodynamic equilibrium when bubbles go into a surrounding gas. Processes of association of bubbles and the process of bubble floating up are favorable processes from the thermodynamic standpoint, because they lead to a decrease of the bubble mechanic energy in accordance with the Laplace formula (3).

## Floating-up of bubbles in liquid

Thus we see that if the amount of a gas in a liquid exceeds the solubility limit of the gas molecules in the liquid, all the excess of this gas forms bubbles in the liquid, provided the amount of liquid is sufficient to contain those bubbles. Hence, evolution of gaseous bubbles in a liquid results in processes of association via contacts between bubbles. Correspondingly, the evolution of a gas in a liquid, with the gas inserted into the liquid in the form of individual molecules or small bubbles, consists in bubble growth and bubble loss by floating-up. Bubbles leave a liquid as a result of the floating-up to the liquid interface. If the distance from a bubble to the interface is *L*, the effective time of bubble floating-up is

9 $$\begin{aligned} \frac{1}{\tau _{fl}}=\frac{v_{fl}}{L}. \end{aligned}$$

The velocity of the floating-up process $v_{fl}$ follows from the balance of the gravitation force acting on a bubble in a liquid and the resistance force. If the resistance force is taken as the Stokes formula [4], this velocity for a small bubble of radius *r* is given by formula

10 $$\begin{aligned} v_{fl}=\frac{2\rho g r^2}{9\eta }, \end{aligned}$$

where $g=980\,\mathrm{cm}^2/\mathrm{s}$ is the free fall acceleration, $\rho$ is the density difference for water and air inside bubbles or the water density; $\eta$ is the water viscosity coefficient. A more careful analysis indicates that the force according to Stokes formula [4], governing motion of a solid particle in a gaseous or liquid medium, must be multiplied by a factor 3/2 [7] if a gaseous or liquid particle is moving in a liquid. Accounting for this, we obtain

11 $$\begin{aligned} v_{fl}=\frac{4\rho g r^2}{27\eta }, \end{aligned}$$

where *r* is the current radius of a test bubble. For an air bubble moving in water at room temperature ($\rho =1\,\mathrm{g}/\mathrm{cm}^3, \eta =8.9\times 10^{-3}\,\mathrm{g}{/}(\mathrm{cm} \, \mathrm{s})$) one can reduce this formula to the form

12 $$\begin{aligned} v_{fl}=C r^2,\quad C=1.6\times 10^4\,\mathrm{cm}^{-1}\,\mathrm{s}^{-1}. \end{aligned}$$

Under these conditions one can represent formula (12) also in the form

13 $$\begin{aligned} v_{fl}=v_on,\quad v_o=1.1\times 10^{-12}\,\mathrm{cm}/\mathrm{s}. \end{aligned}$$

In particular, formulas (9) and (13) give, for bubbles of radius $r=1\,\upmu \mathrm{m}$, the floating-up time $\tau _{fl}=L/v_{fl}=2$ h for air bubbles in water at $L=1$ cm. From this it follows that the lifetime of micron-size air bubbles in quiet water is measured by hours.

## Bubble growth in liquid

**Fig. 2:**
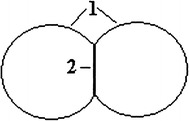
Character of interaction of two contacting bubbles. *1* Interface between a bubble and liquid, *2* film separating bubbles

When bubbles are located in a liquid, they move there and may be in contact as it is shown in Fig. 2. If two bubbles share a mutual boundary (a film 2 in Fig. 2), they cannot fly apart because of the polarization interaction in this system. Therefore the evolution of contacting bubbles may be twofold, so that in the first case a film between bubbles is destroyed and a combined bubble forms, whereas in the second case an amphiphilic film between bubbles is stable and in the end association of multiple bubbles sharing film surfaces leads to formation of a foam. Evidently, the first scenario is realized in simple cases; in particular, it takes place in pure water. This process is analogous to the cluster growth process as a result of association of aerosols [8–10], where liquid spherical particles are located in a gas and their motion in a gas has Brownian character. The size distribution of particles in this case is governed by the Smoluchowski equation [6], and the rate constant of aerosol association is determined by their friction in a gas due to diffusive motion. The classical theory of aerosol growth is suitable also for the growth process of clusters [11, 12] in the diffusion regime.

The second case, in which an amphiphilic film between two contacting bubbles is stable, takes place in organic liquids or if surfactants are added to water that stabilize a film between bubbles. We will consider below the first type of bubble growth and use the appropriate results for cluster growth.

In this regime of bubbles, as a result of their association, growth proceeds according to the scheme

14 $$\begin{aligned} B_n+B_m \rightarrow B_{n+m}, \end{aligned}$$

and each contact of two bubbles leads to their association. The rate of this process is limited by diffusion of gaseous bubbles in a liquid and the rate constant $k_a{s}$ of two bubbles of radii $r_1$ and $r_2$ by analogy with association of two clusters in a dense buffer gas is given by [10–12]

15 $$\begin{aligned} k_{as}=\frac{2T}{3\eta }\left( \frac{r_1}{r_2}+\frac{r_2}{r_1} \right) , \end{aligned}$$

where $\eta$ is the liquid viscosity. We consider the regime of bubble growth in which their size is large compared to the mean free path of liquid molecules in the liquid, and the friction force in motion of bubbles is governed by the liquid viscosity. Then the solution of the Smoluchowski equation [6] that is the kinetic equation for the size distribution function of bubbles has an automodel form [13]. This means that the size distribution function as a function of the ratio of the bubble size to its average size is independent of time, whereas the average bubble size increases in time. Thus, because the process of bubble growth by coagulation of bubbles dominates, it is analogous to cluster growth, and the size distribution function has the form [11, 12]

16 $$\begin{aligned} f_n=\frac{4N_t}{\overline{n}^2}\exp \left( -\frac{2n}{\overline{n}}\right) , \end{aligned}$$

where *n* is the number of air molecules in a bubble, $N_t$ is the total number density of gaseous molecules in bubbles, and $\overline{n}$ is the average number of gaseous molecules in bubbles. One can use an approximation by assuming a relatively narrow distribution function that allows us to replace the factor $(r_1/r_2+r_2/r_1)$ in formula (15) by 2. In particular, in the case of air bubbles in water the rate constant of bubble association at atmospheric pressure and room temperature is equal to

17 $$\begin{aligned} k_{as}=\frac{4T}{3\eta }=6.1\times 10^{-11}\,\mathrm{cm}^3/\mathrm{s}. \end{aligned}$$

On the basis of the analogy with clusters, we obtain the balance equation for the average number of molecules in bubbles $\overline{n}$ as [11, 12]

18 $$\begin{aligned} \frac{d\overline{n}}{dt}=\frac{1}{2}k_{as}N_m, \end{aligned}$$

and the solution of this equation is

19 $$\begin{aligned} \overline{n}=\frac{1}{2}k_{as}N_tt. \end{aligned}$$

**Fig. 3:**
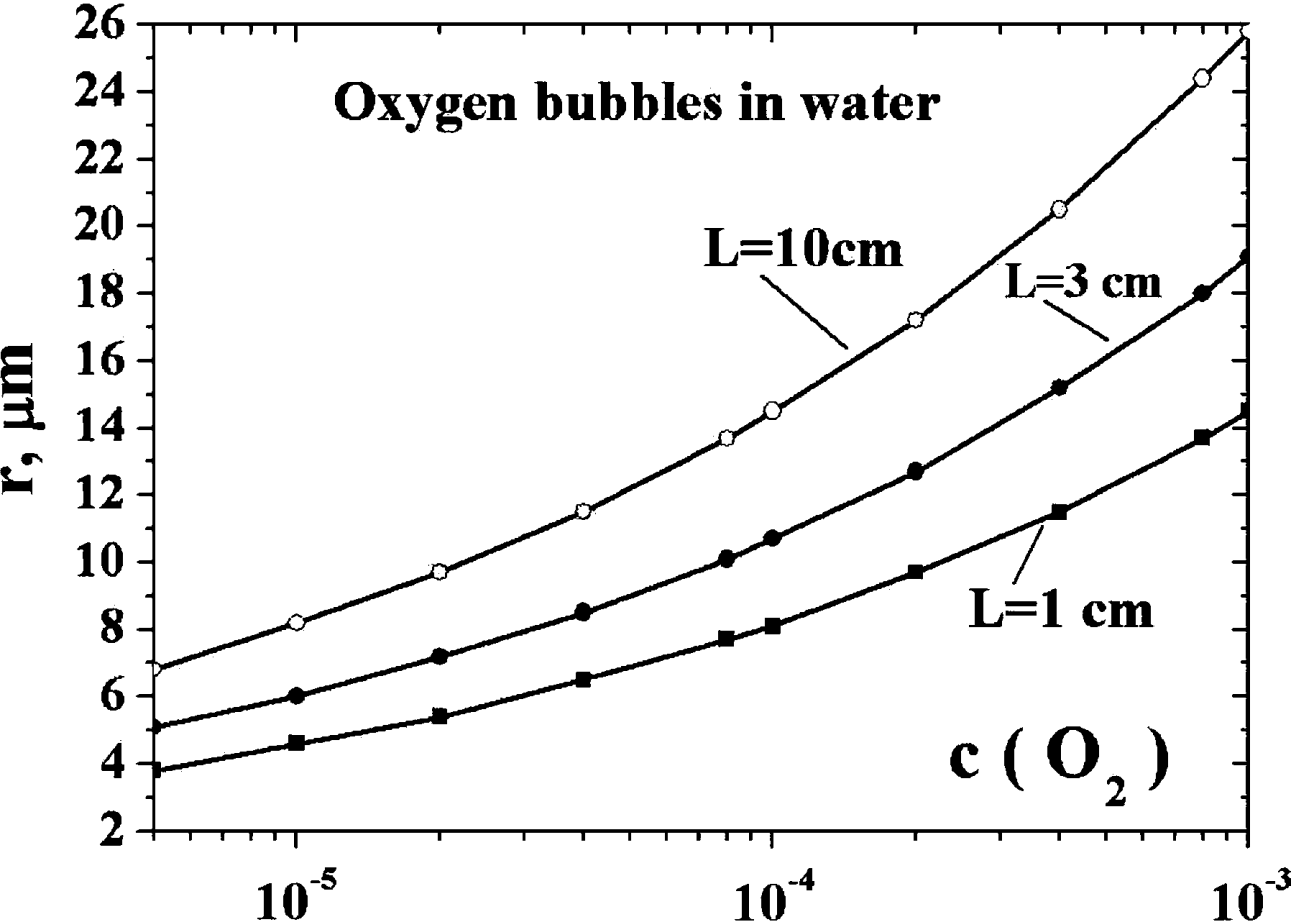
Dependence on the concentration of oxygen molecules in water for an average radius *r* of floating-up bubbles at different initial distances of oxygen from the interface

We assume here that through time *t* after the beginning of coagulation, the average bubble size exceeds its initial size significantly. Hence the size distribution function of bubbles is independent of the initial distribution of the early stage when bubbles formed from individual molecules. Figure 3 gives the parameters of growth of oxygen bubbles in water depending on conditions.

Let us discuss the results given in Fig. 3. Oxygen bubbles are grown as a result of their contacts in the course of Brownian motion, and the growth time decreases with an increase of the oxygen concentration *c* (the ratio of the number of oxygen molecules to the number of water molecules in a certain volume). On the other hand, the velocity of bubble floating up increases witn an increasing size. Participation of both processes gives the results given in Fig. 3.

## Growth of air bubbles in a water container

**Fig. 4:**
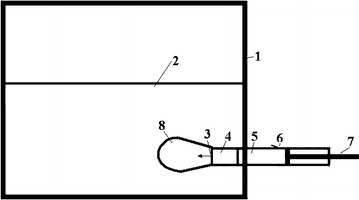
Scheme of injection of submicron air bubbles into water: *1* container, *2* water, *3* flux of air bubbles, *4* porous material, *5* compressed air, *6* valve, *7* plunger, *8* region of high air density

We now apply the above results to certain systems. Let us apply these formulas of bubble growth to conditions of an experiment [3] in which air was inserted in water through a modified porous silica gel. We use a scheme given in Fig. 4 where water is in a container, and air is inserted into the water through a tube with a porous material, so that a heightened air pressure outside the water is supported by a plunger. Percolating through pores, air enters the water in the form of thin air jets whose radii are comparable with the diameter of the pores. In water near the interface with a porous material, each jet breaks up into air bubbles due to the Rayleigh–Plesset [14, 15] instability. Indeed, in the case of pore sizes below $1 \,\upmu \mathrm{m}$, the pressure under the action of the water surface tension exceeds atmospheric, and each individual air jet is compressed and divides into bubbles of smaller sizes. Then the newly-formed bubbles grow according to the scheme (14), as illustrated schematically in Fig. 4. In the first stage of evolution, bubbles spread over a distance of the order of the tube radius, and at this stage of bubble evolution, the total number density of air molecules in bubbles is of the order of the number density of molecules in atmospheric air. In the second stage of evolution, bubbles formed in the first stage spread throughout the water volume. As a result, the rate of bubble generation matches the rate of the floating up process for bubbles, including accounting for their growth due to the coagulation process.

Under these conditions we operate with two typical times; the first one $\tau _1$ is a time during which the total number density of air molecules in bubbles in the course of their evolution is conserved, and the second time $\tau _2$ is the typical duration of an air molecule to be in the total container volume, which is a typical time of floating up for air bubbles located throughout the water volume. In order to understand the real character of bubble evolution, we evaluate parameters of this evolution under conditions of experiment [3]. The tube radius where the porous material is located is $R=0.56$ cm. The flow velocity *V* varies in the experiment [3] from 0.5   to 3.7 m/s. From this we find that the rate of insertion of air molecules into water through a porous material is

20 $$\begin{aligned} J=VN_ts, \end{aligned}$$

where $N_t$ is the number density of air molecules in the flow that enters the water, $s=\pi R^2$ is the cross section of the tube filled by a porous material, and its radius is $R=0.56$ cm in the experiment [3]. The number density of air molecules in bubbles near the entrance into the water is given by

21 $$\begin{aligned} N_t=\epsilon N_a, \end{aligned}$$

where we take the porosity coefficient $\epsilon =0.57$ according to experimental conditions, and the number density of air molecules in pores $N_a$ near the entrance of jets into the water we take to be that at atmospheric pressure. This gives $N_t=1.4\times 10^{19}\,\mathrm{cm}^{-3}$, and under experimental conditions the rate of injection of air molecules into water through the porous material ranges from $J=7\times 10^{20}\,\mathrm{s}^{-1}$ up to $J=5\times 10^{21}\,\mathrm{s}^{-1}$ or from 0.03 to 0.2 g/s depending on the velocity of jets at the water boundary.

As we discussed above, we divide the evolution of bubbles in water into two stages. In the first stage, the flow of water forming bubbles does not include new water additions, i.e. the number density of air molecules in this flow $N_t$ does not vary, although bubbles grow. In the second stage of bubble evolution, they mix with water in the container. This leads to a decrease in the total number density of air molecules in the container, and we assume that bubbles mix with water of the container uniformly. We assume that the number density of air molecules in bubbles does not vary so long as the flowing bubbles do not encounter container walls. This proceeds during a time $\tau =d/V$, where $d=10$ cm is the experimental container diameter, and under experimental conditions this time varies from 0.03 to 0.2 s, and according to formula (19) the average bubble content ranges from $1.1\times 10^7$ to $8.4\times 10^7$ air molecules per bubble. This yields an average bubble radius from 0.3 to $0.8 \,\upmu \mathrm{m}$ which correspond to the measured values [3] (0.25–0.75 $\upmu \mathrm{m}$).

It should be noted that the container volume is $\Omega =3 \times 10^3\, \mathrm{cm}^{-3}$ under experimental conditions [3], and the volume $\Omega _1=sl \approx 10\,\mathrm{cm}^{-3}$ relates to the region 8 of Fig. 3 where the initial number density of air molecules in bubbles is conserved. Correspondingly, the average number density $N_w$ of air molecules in bubbles over all the container is

22 $$\begin{aligned} N_w=N_t \times \frac{\Omega _1}{\Omega }=4.6\times 10^{16}\,\mathrm{cm}^{-3}. \end{aligned}$$

If these air molecules constitute bubbles of radius $1\,\upmu \mathrm{m}$, this corresponds to the number density $N_b=3.6\times 10^8\,\mathrm{cm}^{-3}$, and bubbles occupy 0.15 % of the water volume. As bubbles evolve in a water reservoir, they grow by coagulation and leave when they reach the water-air boundary. But because motion of water has a turbulent character under experimental conditions [3], departure of bubbles is determined primarily by parameters of turbulent motion, rather than simply by the rate of the smooth floating up of bubbles.

In considering the above regime of the kinetics of bubbles of micron and submicron size, we were guided by experimental conditions [3] in which a gas is injected into a liquid through a porous material with nano-size pores. There, the gas enters the liquid in the form of jets whose radii correspond to pore sizes, and then the jets are destroyed due to the Rayleigh–Plesset instability [14, 15] and transform into micron-size bubbles which then join to bubbles of larger sizes. In particular, within the framework of experiment [3], bubbles that form in water after association of injected bubbles contain a few of hundreds of the initially injected bubbles.

## Liquid flow with bubbles in tube

Let us consider one more example, in which bubbles propagate along a tube in a liquid flow. In this example we will be guided by the blood flow [16–18]. It should be noted that if contrast agents or drugs are injected in blood, they dissolve in blood water, and just this mixture is used in clinical applications [19–21]. Therefore an injected medicine must not react with walls of a blood vessel in this case. In the case under consideration an injected substance may react with a blood vessel, and it is necessary to transport the medicine through a blood vessel without contacts with walls. Let us inject this medicine in the center of a blood flow in the form of micron-size bubbles or droplets and they travel over the vessel cross section as a result of diffusion or floating-up. For example, these bubbles may contain ozone or another oxidizer, and these substances lose chemical activity if bubbles contact the walls of a blood vessel. Below we determine the lifetime of bubbles in a blood flow under these conditions.

Being guided by a venous duct, we take the blood velocity of 10–15 cm/s and a duct radius of 3–4 mm, so that a bubble residence time in a venous duct does not exceed $\tau _r \sim 10\,\mathrm{s}$. Because the blood consists of $90\,\%$ water by volume, one can model blood flow in a blood vessel as a water flow. Comparing the propagation of a bubble in water due to floating-up and diffusion, one obtains that transport due to floating-up takes place for distances *L*

23 $$\begin{aligned} L \gg \frac{4D_b}{v_{fl}}, \end{aligned}$$

where $D_b$ is the diffusion coefficient of bubbles in water, and the floating-up velocity $v_{fl}$ is given by formula (11). The diffusion coefficient of nitrogen molecules in water at room temperature is $D(N_2)=2.0 \times 10^{-5}\,\mathrm{cm}^2/\mathrm{s}$ [5], and for oxygen molecules this value is $D(O_2)=2.4 \times 10^{-5}\,\mathrm{cm}^2/\mathrm{s}$ [5]. The air molecules in a flow move due to their diffusion and for the residence time of a blood flow $\tau _r$, the transverse propagation $\Delta$ of air molecules is given by

24 $$\begin{aligned} \Delta \sim \sqrt{4D\tau _r}\sim 0.3\,\mathrm{mm}, \end{aligned}$$

this distance is small compared to the blood vessel radius.

The diffusion coefficient for a micron-size bubble in water at the temperature $T=300$ K is given by [11, 12]

25 $$\begin{aligned} D_b=\frac{D_o}{r},\quad D_o=2.5\times 10^{-11}\,\mathrm{cm}^3/\mathrm{s}. \end{aligned}$$

Taking $L=3$ mm for a venous duct and using formulas (11) and (25) for the floating-up velocity and diffusion coefficient of air bubbles in water, one can obtain the criterion (23) to be valid at $r \gg 0.3\,\upmu \mathrm{m}$. Hence, the floating-up mechanism is realized for bubble transport of micron-size bubbles through a blood vessel. In this process the bubble displacement $\Delta = v_{fl}\tau _r$ during the residence time of bubbles must be small compared to the blood vessel radius, which leads to the criterion for the bubble radius

26 $$\begin{aligned} r \ll 40\,\upmu \mathrm{m}. \end{aligned}$$

If this criterion holds true, the bubble displacement in blood flow through a venous duct is small compared with its radius. Along with this criterion, there is a requirement of a restricted number density of bubbles, so that a doubling time of a bubble size as a result of their association must exceed their residence time in the flow. In particular, in the case under consideration for bubbles of a radius $r=1\,\upmu \mathrm{m}$, the total concentration $c_m$ of bubble air molecules in water according to this requirement and the rate of bubble association (18) is $c_m \ll 3\times 10^{-5}$.

In addition, one can take instead of bubbles micron-size droplets. These bubbles and droplets may be used not only for transport of some substances through a blood vessel, but they can be catalysts to remove harmful compounds from the blood. Above we analyze conditions where transport of bubbles through a blood vessel excludes their interaction with vessel walls. Another problem of their interaction with red corpuscules will require a special analysis of its own.

## Conclusion

Disperse systems under consideration consist of a liquid and gaseous bubbles. If a gas is inserted into a liquid and its amount exceeds the maximum level of solubility of that gas, the excess gas forms bubbles in the liquid through time. Processes in such a system with micron-size bubbles include diffusion and floating-up of bubbles, as well as association of contacting bubbles. The above rates of these processes allow us to describe the kinetics of growth for a disperse system of such a type.

We consider disperse systems located in a container with an open surface containing the gas above the interface. Because of the long time of bubble floating-up under laboratory conditions, bubbles grow in the liquid and their size is independent of initial conditions when they reach the interface. This fact simplifies the problem and allows us to connect a size of floating-up bubbles with the gas concentration in the liquid and the path to the interface. The above analysis allows one to choose optimal conditions for any process involving micron-size bubbles or droplets for a certain disperse system.

In analyzing the behavior of a gas inside a liquid, we show that thermodynamic equilibrium in this system takes place between dissolved molecules and the liquid, while excess molecules leave the liquid. In the regime under consideration an outgoing time is relatively long, and the number density of dissolved molecules is small compared to undissolved ones, and this gas-liquid system is a nonequilibrium one. In the end, undissolved molecules leave the liquid through its boundary, and most of the time in the course of this process, undissolved molecules are in the liquid in the form of bubbles. Then the rate of exit of undissolved gas molecules in the liquid is summed from kinetics of bubble growth as a result of their association and floating up under the action of the gravitation force. We use here the classical theory of growth kinetics of aerosols in a gas [8–10] which is constructed on the basis of the Smoluchowski equation [6] and was applied to growth of liquid clusters in a gas [11, 12] in the diffusion regime of the cluster growth process. Using the analogy of the above growth processes with bubble growth in a liquid, we apply formulas for kinetics of aerosol and cluster growth in this case. As a bubble grows, its floating velocity increases, and accounting for both processes allows us to determine the bubble life time in a liquid with an open upper surface. The evaluations are made for the growth and floating up of the oxygen bubbles in water. This process takes place when oxygen results for the photosysntesis process in a water reservoir.

It should be noted that this model may be used also for the analysis of bubble evolution of liquid flows if they propagate through a tube. This may be used for injection of drugs or contrast agents in blood that propagates through a blood vessel [16–18]. In such applications, usually the injected substance is mixed with a blood or is dissolved in it [19–21]. The above approach allows one to deliver a substance in the form of bubbles or droplets, and the above theory gives conditions to escape interaction of injected bubbles with vessel walls. This provides a list of possible medical applications.
